# Heparanase attenuates axon degeneration following sciatic nerve transection

**DOI:** 10.1038/s41598-018-23070-6

**Published:** 2018-03-26

**Authors:** Michael J. Whitehead, Rhona McGonigal, Hugh J. Willison, Susan C. Barnett

**Affiliations:** Institute of Infection, Immunity and Inflammation, College of Medical, Veterinary and Life Sciences, University of Glasgow, 120 University Place, Glasgow, G12 8TA UK

## Abstract

Axon degeneration underlies many nervous system diseases; therefore understanding the regulatory signalling pathways is fundamental to identifying potential therapeutics. Previously, we demonstrated heparan sulphates (HS) as a potentially new target for promoting CNS repair. HS modulate cell signalling by both acting as cofactors in the formation of ligand-receptor complexes and in sequestering ligands in the extracellular matrix. The enzyme heparanase (Hpse) negatively regulates these processes by cleaving HS and releasing the attached proteins, thereby attenuating their ligand-receptor interaction. To explore a comparative role for HS in PNS axon injury/repair we data mined published microarrays from distal sciatic nerve injury. We identified Hpse as a previously unexplored candidate, being up-regulated following injury. We confirmed these results and demonstrated inhibition of Hpse led to an acceleration of axonal degeneration, accompanied by an increase in β-catenin. Inhibition of β-catenin and the addition of Heparinase I both attenuated axonal degeneration. Furthermore the inhibition of Hpse positively regulates transcription of genes associated with peripheral neuropathies and Schwann cell de-differentiation. Thus, we propose Hpse participates in the regulation of the Schwann cell injury response and axo-glia support, in part via the regulation of Schwann cell de-differentiation and is a potential therapeutic that warrants further investigation.

## Introduction

Understanding the mechanisms underlying Wallerian degeneration in the peripheral nervous system (PNS) has progressed significantly since the discovery of the Wld^s^ mouse^1^, which notably delays axonal degeneration in the distal stump after transection injury. Crossing the Wld^s^ mice with various peripheral neuropathy models also leads to significant axonal protection^2–4^. The contribution of downstream pathways to axon degeneration are currently the subject of investigation, and include mitochondrial dysfunction, disruption of energy supply, calcium homeostasis and the activation of calpain proteases^5–8^. Schwann cells also play important roles in repair including phagocytosis of debris, promotion of regeneration via their secretome and ultimately, remyelination of regenerating axons^9^. There is no known direct causative link between Schwann cells and axon degeneration after injury, although it is becoming increasingly clear that Schwann cells provide metabolic support to axons and their disruption is associated with axonal degeneration^10–12^.

We have previously shown that heparin sulphate mimetics can modify certain characteristics of reactive astrocytosis^13,14^. Furthermore, treatment of dorsal root ganglion (DRG) cultures with heparin promotes PNS myelination *in vitro* via the specific inhibition of the soluble immunoglobulin (Ig)-containing isoforms of neuregulin 1 (i.e., NRG1 types I and II), that negatively regulate myelination^15^. Herein we hypothesised that heparan sulphates (HS) could also modulate the PNS injury response. HS are linear glycosaminoglycan (GAG) chains attached to proteoglycans (HSPG). They are found in the extracellular matrix, attached to the extracellular leaflet of the plasma membrane. HS play an important role in the regulation of many cellular functions^16,17^ including facilitation of the binding of proteins to their corresponding receptors, such as FGF and Wnt ligands^18–21^. HS mediated signalling is regulated by enzymes that cleave them, such as the endoglycosidase heparanase (Hpse), which release bound proteins^22^. Hpse acts both at the cell-surface and within the extracellular matrix to degrade polymeric heparan sulfate molecules into shorter chain length oligosaccharides. Hpse is a prognostic marker for poor outcome in malignant tumours and its activity correlates with inflammatory acidosis^23^. Increased Hpse expression is also being increasingly implicated in diseases associated with neuroinflammation^24^. Whilst Hpse expression has been identified in astrocytes and neurons of the CNS and has been shown to promote neurite extension *in vitro*^25–27^, there is currently, to the best of our knowledge, no association with Schwann cells or the PNS injury response.

In this study we have data mined previously published microarrays generated after sciatic nerve injury to identify genes associated with changes in HS. We identified the enzyme Hpse, known to degrade HS, as an mRNA upregulated after injury, in the distal stump. To investigate the biological relevance of Hpse activation following PNS injury we used two models: an *ex vivo* model of sciatic nerve injury and a nerve-muscle preparation which models distal motor nerve terminal injury. We identify an increase in Hpse after injury and accelerated axon loss plus myelin basic protein (MBP) loss correlating with Schwann cell de-differentiation following Hpse inhibition. This occurs at least in part due to Hpse’s ability to regulate β-catenin and subsequently Sox2 transcription.

## Results

### Hpse activity is consistently up-regulated after distal SN injury

**Figure 1:**
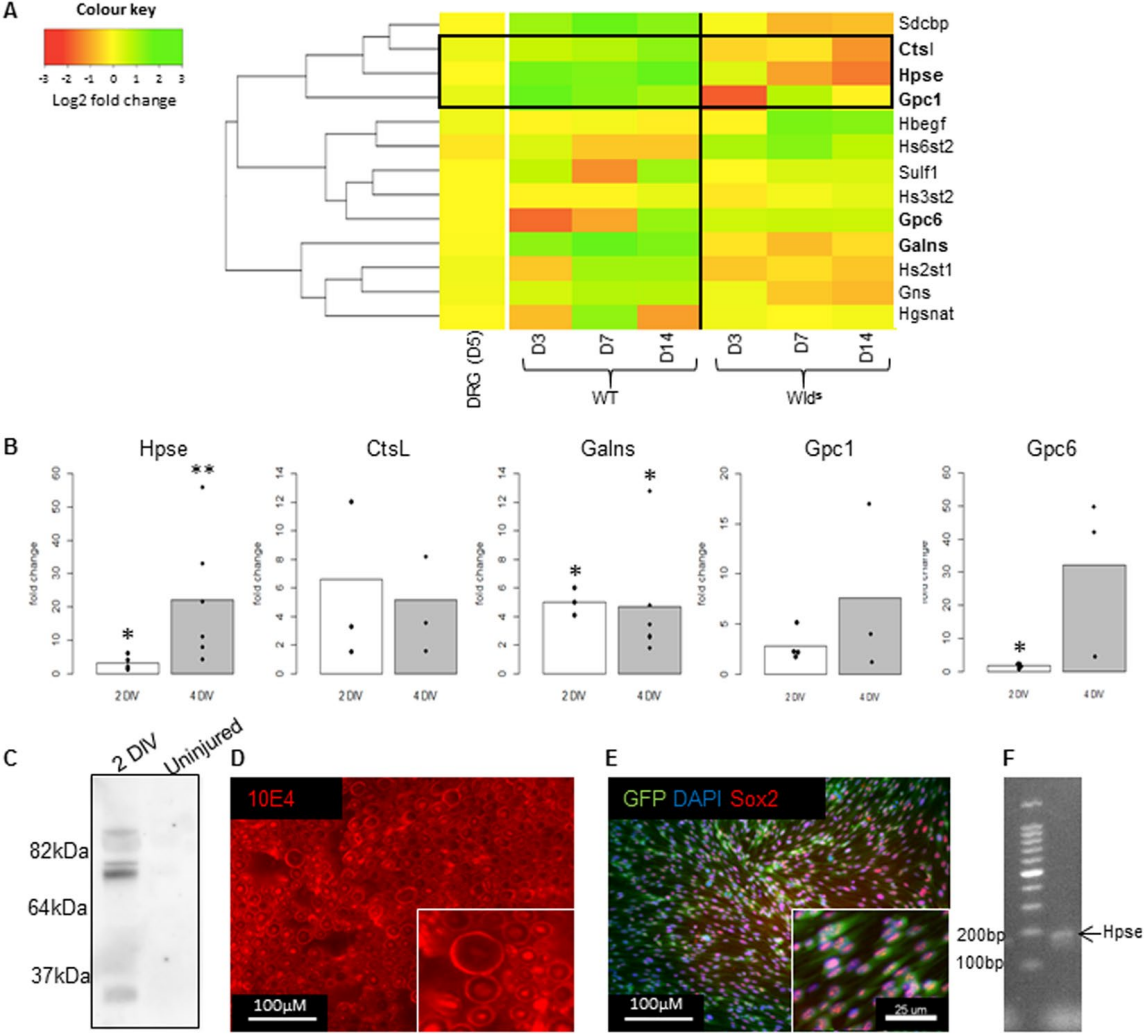
Microarray, qPCR and protein analysis indicates a role for HS mediated signalling in Wallerian degeneration. (**A**) Heat map to show microarray analysis from two different published array sets obtained from GEO (refer to methods) for HSPG genes as well as genes associated with HS biosynthesis and regulation. Genes coloured yellow are not differentially expressed (DE) while genes with increasing expression become a gradually darker green, conversely genes that decrease in expression become gradually darker in orange and then red. The dendrogram, which illustrates clustering between genes, used a Euclidean measure to calculate distance and the complete agglomeration method for clustering, according to the heatmap.2 R package. Clustering of Ctsl, Hpse and Gpc1 is indicated by the box. Vertical bold line separates the profile from WT (left) and Wld^s^ (right) mice. D = day. (**B**) RT-qPCR validation of selected highly expressed genes from the microarray results using an *ex vivo* SN injury model confirms Hpse is highly (significantly) up-regulated. All results compare uninjured SN to *ex vivo* SN after either 2 or 4 DIV. Hpse (2 DIV - *p = 0.03, n = 5, 4 DIV – **p = 0.0009, n = 6). Ctsl (2 DIV - p = 0.2, n = 3, 4 DIV – p = 0.12, n = 3). GALNS (2 DIV – *p = 0.043, n = 3, 4 DIV – *p = 0.006, n = 6). E) Gpc1 (2 DIV - *p = 0.06, n = 4, 4 DIV – p = 0.22, n = 3) Gpc6 (2 DIV – *p = 0.04, n = 4, 4 DIV = p = 0.059 n = 3). (**C**) Representative full Western blot from one experiment of, *ex vivo* SN, illustrates an upregulation in immunoreactivity for 3G10 antibody, that detects HS cleaved by Hpse, after 2 DIV (injured) compared to uninjured control – where no immunoreactivity was detected (n = 3). Amido black whole protein stain was used for loading control (refer to supplementary section Fig. S4). (**D**) Immunoreactivity/fluorescent staining for the antibody IOE4, which recognises a wide array of HS expression, demonstrates baseline presence of HS in the uninjured SN. Scale bar = 50 μM. (**E**) Immunostaining for Sox2 and DAPI in purified rat Schwann cells expressing endogenous GFP shows that (**F**) Hpse is expressed in purified rat Schwann cells using PCR (n = 2). Students T test or one sample T test was used for all statistical analysis.

To investigate a role for HS in the PNS we first analysed three previously published microarray (mRNA) data sets; two for distal SN injury and one for the DRG after SN injury. Figure 1A shows a heat map of the expression of HSPGs, as well as their biosynthetic and signal regulating enzymes derived from two microarray datasets. One comprised of data from transected SN from WT and Wld^s^ mice at 3, 7 and 14 days (GSE22291) and the second comprised data from the DRG after injury (GSE58982). The Wld^s^ mouse significantly delays axon degeneration after SN transection and is therefore a useful comparison to WT mice for identifying mechanisms associated with axon degeneration. In these datasets no significant difference in expression (DE) is observed for HSPGs, except for glypican-1 (Gpc1) and glypican-6 (Gpc6). Upregulation of Hpse was seen after WT injury at all time points, and its gradual down-regulation was observed following injury in in Wld^s^ compared to WT (Fig. 1A). A further published microarray for the injured distal SN also showed upregulation of Hpse (7 days after injury; GSE38693)^28^. Cathepsin–L (Ctsl), which activates Hpse enzymatic activity^29^, showed a similar expression profile to Hpse and the cluster analysis groups Hpse, Ctsl and Gpc-1 together (Fig. 1A). Galactosamine (N-acetyl)-6-Sulfatase (GALNS), which is up-regulated after WT injury and comparatively decreased in Wld^s^ injury, also removes 6-O sulphate groups^30^. To verify the published microarray results we first used an *ex vivo* model of SN injury^31^. The advantages of using this model over whole animal *in vivo* SN injury include the ability to isolate the SN from the infiltrating immune system, ease of inhibitor administration and reduced animal suffering. RT-qPCR after 2 and 4 DIV confirmed Hpse (3/15 fold, p = 0.03/0.0009) and GALNS (5/4.67 fold, p = 0.043/0.006) were significantly up-regulated compared to uninjured control (Fig. 1B). Changes in Ctsl, Gpc1 and Gpc6 were not statistically significant after injury; however there was a trend towards an increase in expression (Fig. 1B).

To demonstrate an increase in Hpse activity after SN injury we used the antibody 3G10, which binds to HS stubs cleaved by Hpse, thereby acting as indirect evidence for heparanase activity. Western blot analysis of the *ex vivo* SN confirmed an increase in 3G10 antibody binding after 2 DIV, when compared to the uninjured SN (Fig. 1C). Figure 1D shows immunoreactivity (IR) for the antibody 10EA, which binds most HS, in the uninjured SN. This illustrates the presence of HS both axonally, and on the abaxonal layer of the myelin sheath (Fig. 1C). As Schwann cells are the predominant cell type in the SN we investigated whether Schwann cells express Hpse by PCR. Using purified cultures of Schwann cells we detected a band at the expected size for Hpse (Fig. 1E,F), suggesting that in the *ex vivo* sciatic nerve tissue, Hpse is expressed at least by Schwann cells.

### Hpse protects against acute axonal loss in the *ex vivo* sciatic nerve

**Figure 2:**
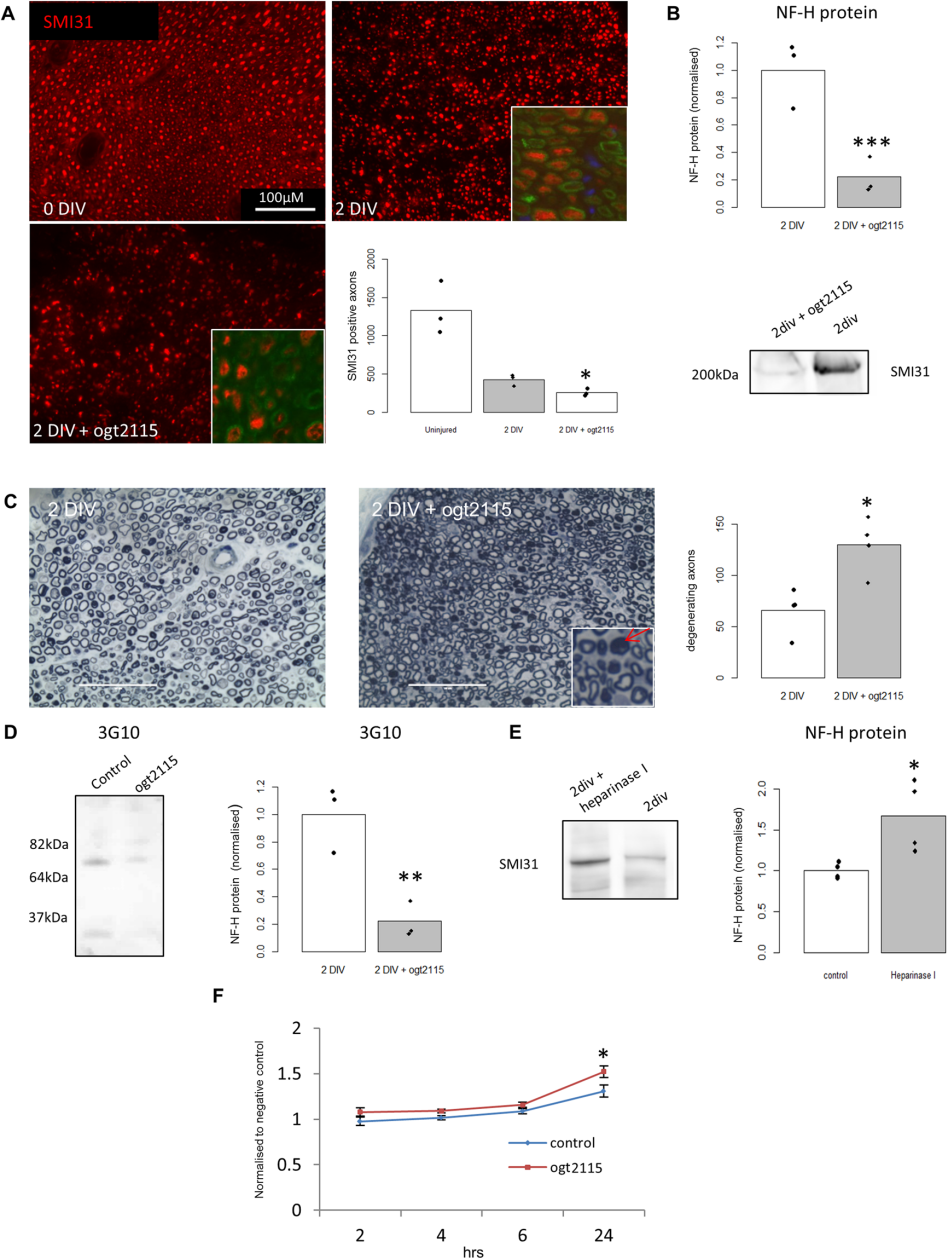
Heparanase inhibition accelerates acute axonal degeneration in *ex vivo* sciatic nerves. (**A**) Representative images of SMI31-IR (red) immunostaining of sections from *ex vivo* SN cultures either uninjured (naïve), after 2 DIV or 2 DIV treated with OGT2115, the Hpse inhibitor. In magnified images; MBP-IR (green) and DAPI-IR (blue). Quantification of SMI31-IR axons of images, *p = 0.02, n = 3. (**B**) Representative cropped Western blot from one experiment, for *ex vivo* SN, (representative full length blot is available in supplementary section Fig. S4) after OGT2115 treatment showing a significant decrease in SMI31 expression, quantified by densitometry, ***p = 0.0002, n = 3. Amido black whole protein stain was used for loading control (representative blot available in supplementary section Fig. S4). (**C**) Semi-thin sections of resin embedded *ex vivo* SN after 2 DIV or 2 DIV with OGT2115 treatment stained with methylene blue/azurII. Quantification of degenerating axons in the *ex vivo* SN after 2 DIV treated with OGT2115 showed a significant increase compared to 2 DIV alone, *p = 0.012, n = 4. Red arrow indicates a collapsed myelin sheath which represents a degenerating axon. (**D**) Representative Western blot of *ex* vivo SN after 2 DIV with or without OGT2115 showing a significant decrease in HS (3G10) cleaved by Hpse **p = 0.008, n = 3. (**E**) Representative cropped Western blot from one experiment, of *ex vivo* SN, (representative full length blot is available in supplementary section Fig. S4) after 2 DIV with or without Heparinase I treatment showing a significant decrease in the loss of SMI31 protein levels *p = 0.026, n = 3. Amido black whole protein stain was used for loading control (representative blot available in supplementary section Fig. S4). (**F**) Alamar blue analysis for cell viability of *ex vivo* SN treated with OGT2115. It can be seen that there was no difference in levels between OGT2115 treatment and control (not shown) until 2 DIV where a significant increase was observed after OGT2115 treatment, *p = 0.018. n = 3. The data from the alamar blue assay was normalised to results obtained from cultured media lacking cells containing the alamar blue only, which is defined as the negative control. Students T test was used for all statistical analysis or one-way ANOVA with post hoc Tukey HSD test.

In order to determine the role of Hpse in distal SN degeneration we treated the *ex vivo* SN with the Hpse inhibitor OGT2115. Quantification of SMI31-IR positive axons in nerve sections showed a 40% acceleration of axon loss with OGT2115 treatment, after 2 DIV (Fig. 2A, p = 0.02). We selected 2 DIV since after peripheral nerve injury it has been reported that there is an additional latency phase before axon degeneration takes place, which is marked at approximately 36–40 hrs in mouse *ex vivo* sciatic nerve injury^10^. Western blot analysis of the tissue (Fig. 2B) also showed a 90% (p = 0.0002) decrease in SMI31 protein levels after 2 DIV with OGT2115 treatment. Consistent with this, quantification of degenerating axons revealed a two fold increase after OGT2115 treatment compared to 2 DIV alone (Fig. 2C) (p = 0.012). The 3G10 anti-HS stub antibody was then used to assess whether OGT2115 was inhibiting Hpse. Western blot analysis of the tissue (Fig. 2C) reveals a 78% reduction in 3G10 binding (p = 0.008). Addition of heparinase I (a substitute for Hpse)^32^ caused a 70% attenuation of the loss of SMI31 protein levels in the *ex vivo* SN after 2 DIV as indicated by Western blot (Fig. 2E, p = 0.026). It was not possible to test heparinase I treatment on axonal degeneration *ex vivo* as experiments suggest the enzyme was not able to sufficiently penetrate the tissue (data not shown). Alamar blue, a redox indicator that yields a colorimetric change and a fluorescent signal in response to metabolic activity was used to confirm that OGT2115 treatment was not cytotoxic. No difference in signal after alamar blue treatment was found in the *ex vivo* SN with OGT2115 treatment, compared to control, until 2 DIV at which point there was a significant increase in metabolic activity due to the reduction of alamar blue (Fig. 2F).

### Hpse inhibition promotes NMJ injury, in an *ex vivo* distal motor nerve injury model

**Figure 3:**
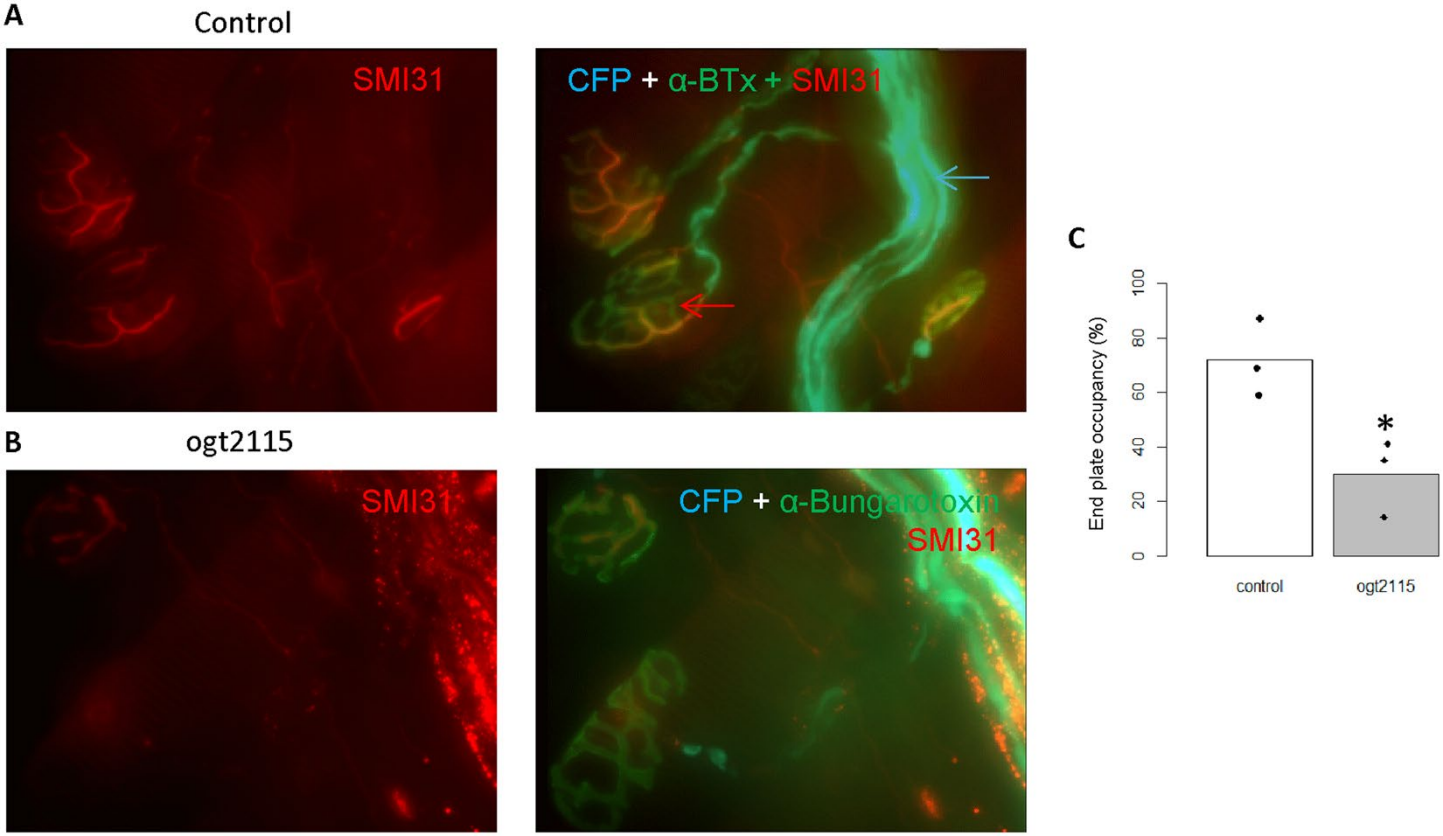
Hpse inhibition promotes loss of innervated NMJ, in an *ex vivo* distal motor nerve injury model. (**A**) Untreated triangularis sterni preparation at 24 h shows normal distal nerve terminal innervation (SMI31-IR (red), CFP expression (cyan) in axons innervating nerve terminals, indicated by ά – bungarotoxin-IR (green). Red arrow indicated end plates. Blue arrow indicates CFP positive intramuscular nerve bundles. (**B**) OGT2115 treated TS preparation shows loss of SMI31-IR (red) and CFP expression (cyan) in axons innervating nerve terminals, indicated by α – bungarotoxin-IR (green). (**C**) Quantification of % SMI31-IR end plate occupancy shows a loss of SMI31-IR end plate occupancy after OGT2115 treatment, *p = 0.025, n = 3.

To further support a role for Hpse activity in enhancing distal peripheral nerve axon survival following axotomy, we used an *ex vivo* nerve muscle preparation from the triangularis sterni (TS) muscle. In this preparation, terminal axons can be seen to project into the neuromuscular junction (NMJ) and be observed to degenerate following proximal axonal injury. Staining allows us to quantify injury as end plate occupancy as defined in the methods. After 24 hrs the ratio of occupied endplates decreased by 70% after OGT2115 treatment, compared with a decrease of 28% in controls (Fig. 3, p = 0.025). This 24 hr time point was the earliest at which changes in the NMJ or signs of injury were seen.

### Hpse down-regulates β-catenin and β-catenin inhibition protects against axonal loss

**Figure 4:**
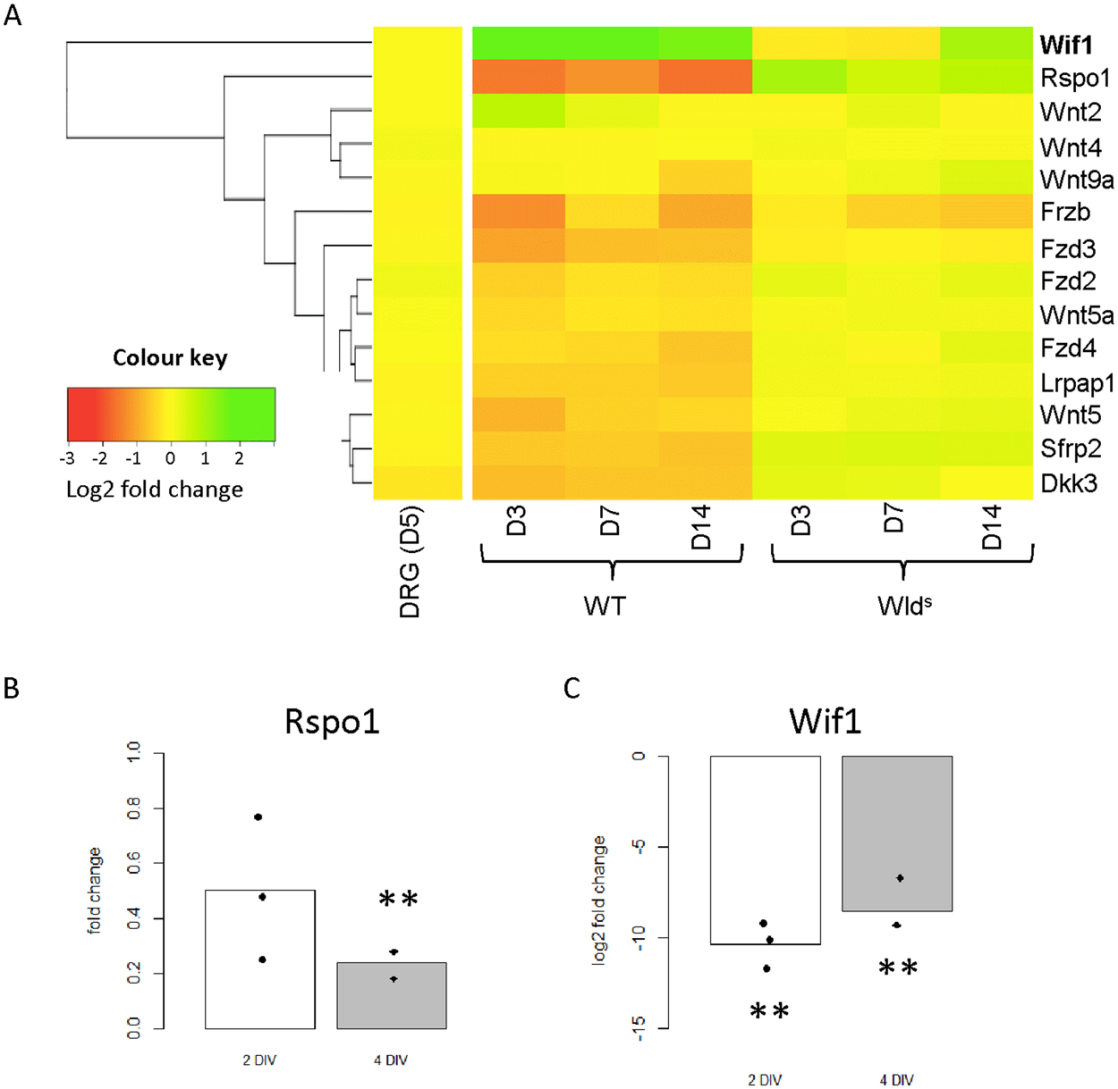
Bioinformatics of DE Wnt signalling components in the distal sciatic nerve after injury shows a decrease in Wnt signalling. (**A**) Heatmap for numerous components of the Wnt signalling pathway. The legend shows that genes not DE are yellow while those that increase in expression become gradually more green and those that decrease in expression becoming gradually more orange and then red. The dendrogram, which illustrates clustering between genes, used a Euclidean measure to calculate distance and the complete agglomeration method for clustering. Wif1 was strongly up-regulated while Rspo1 was down-regulated. D = days. (**B**) RT-qPCR for Rspo1 after 2 and 4 DIV from the *ex vivo* SN shows a significant decrease after 4 DIV (2 DIV – p = 0.089, n = 3, 4 DIV - **p = 0.003, n = 3). (**C**) RT-qPCR for Wif1 after 2 and 4 DIV from the *ex* vivo SN showing the opposite from bioinformatics data (2 DIV – **p = 0.005, n = 3, 4 DIV - **p = 0.0076, n = 3). One sample T test was used for all statistical analysis.

HS are known to facilitate Wnt receptor binding^19^, prompting us to analyse Wnt signalling pathways in SN injury, using the same previously published microarrays. Figure 4A shows a heat map for Wnt ligands, receptors and Wnt signalling regulators. This shows a decrease in expression of Rspo1, Frzb, Fzd3 and Wnt5 and an increase in the expression of Wif1 and Wnt2 (Fig. 4A) in WT injury. When comparing WT injury to Wld^s^ there is a significant decrease in Wif1 and increase in Rspo1 at later time points. Wif1 and Rspo1 are potent regulators of Wnt signalling and are also the most highly differentially expressed (DE) in the published microarrays. Prompting us to validate their differential expression using RT-qPCR in the SN injury model. However while this downregulation was confirmed for Rspo1 (2 DIV – p = 0.089, n = 3, 4 DIV - **p = 0.003, n = 3), Wif1 was also unexpectedly down-regulated (2 DIV p = 0.005, 4 DIV p = 0.0076) (Fig. 4B,C) compared to uninjured control, indicating a discordance with the microarray analysis.

**Figure 5:**
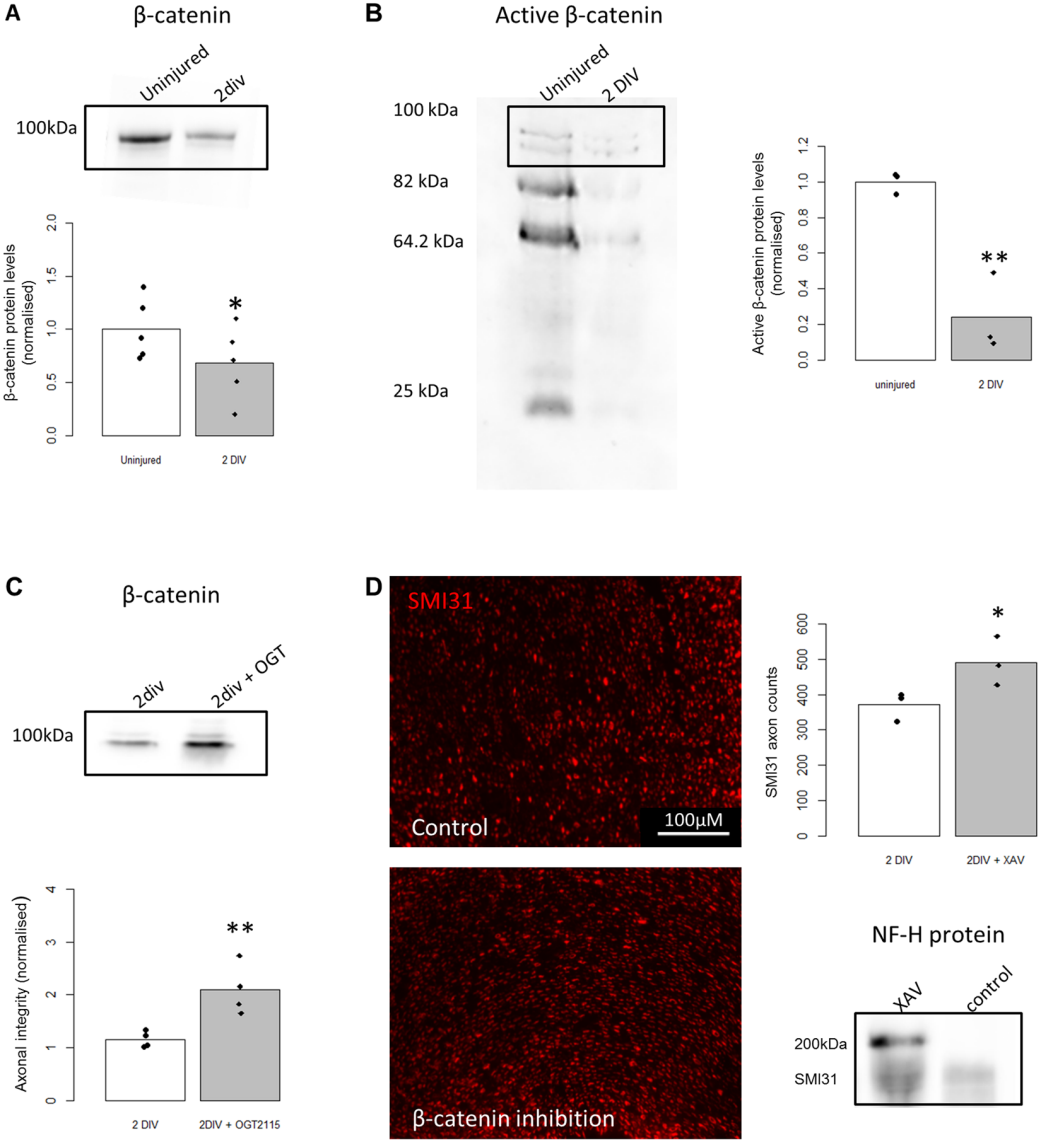
Hpse inhibition in *ex vivo* sciatic nerves increases β-catenin protein levels and β-catenin inhibition protects against SMI31 loss. (**A**) Representative cropped Western blot from one experiment for β-catenin 2 DIV after injury compared to uninjured *ex vivo* SN, showing a significant decrease in β-catenin after injury (n = 5, *p = 0.03). (**B**) Representative cropped Western blot from one experiment for active β-catenin in *ex vivo* SN either uninjured or after 2 DIV. Quantification shows a significant decrease in active β-catenin after injury (n = 3, **p = 0.004). (**C**) Representative cropped Western blot from one experiment for β-catenin in *ex vivo* SN either 2 DIV or 2 DIV with OGT2115 treatment shows a significant increase in β-catenin with OGT2115 treatment (n = 4, **p = 0.009). (**D**) There is a significant protection of SMI31-IR axons and protein levels after 2 DIV with β-catenin inhibition (XAV939 treatment). Images of *ex vivo* SN sections immunolabelled with SMI31 (red) showing axons after 2 DIV with or without XAV939 treatment (n = 3, *p = 0.03). Representative cropped SMI31 Western blot from one experiment for *ex vivo* SN after 2 DIV with or without XAV939 shows protection of SMI31 after XAV939 treatment (n = 3). For all Western blots shown in (**A**–**D**), the full length blots are available in supplementary section (Fig. S4). In all cases amido black whole protein stain was used as a loading control, representative blots are available in supplementary section (Fig. S4). Student T test was used for all statistical analysis.

As an alternative pathway to verify the inhibition of Wnt signalling we next investigated β-catenin, a downstream component of canonical Wnt signalling, by measuring β-catenin protein levels under injured and Hpse inhibitor conditions in the *ex vivo* SN. When comparing uninjured nerves to 2 DIV there was a significant decrease in both β-catenin (Fig. 3B, p = 0.03) and active β-catenin (Fig. 3B, p = 0.004). However after OGT2115 treatment there is a significant two fold increase in β-catenin, compared to 2 DIV alone (Fig. 5C, p = 0.009). β-catenin was then inhibited with XAV939. Quantification of SMI31-IR axons in sections after XAV939 treatment, showed a significant 36% protection of SMI-31-IR axons (Fig. 5D, p = 0.03). Western blot analysis also showed a significant protection of SMI31 protein levels after XAV939 treatment (Fig. 5D).

### Bioinformatics identified β-catenin transcription targets associated with nerve injury response

To further understand signalling induced by the SN injury response we next conducted a more unbiased approach for identifying genes potentially involved in the Schwann cell injury response, again by interrogating published microarrays. A gene was selected if it was differentially expressed only in WT mouse SN injury and not in the Wld^s^ mouse or the DRG after injury (Supplementary Fig. 1). This procedure identified 131 genes, which were then narrowed down to 71 genes by looking at the earliest time point (3 days) to try and identify initial molecular mechanisms. Within this list, genes associated with peripheral neuropathies were enriched. Although these genes were present in the original differentially expressed gene list, their identity was emphasised by this process (Annokey, a high throughput Pubmed search)^33^. Out of these 71 genes, 10 (10/71 genes) are associated with peripheral neuropathies compared to all DE genes (3 days after injury) which have 15 (15/1766 genes).

Furthermore, of these 71 genes, 21 are predicted to be positively transcriptionally regulated by LEF1 and TCF3, which are part of the β-catenin signalling pathway^34^ (Supplementary Fig. 2). In conclusion, we show that β-catenin, which can be negatively regulated by Hpse activity, is predicted to transcriptionally up-regulate 21 genes hypothesised to play a role in SN injury using an unbiased microarray screen.

**Figure 6:**
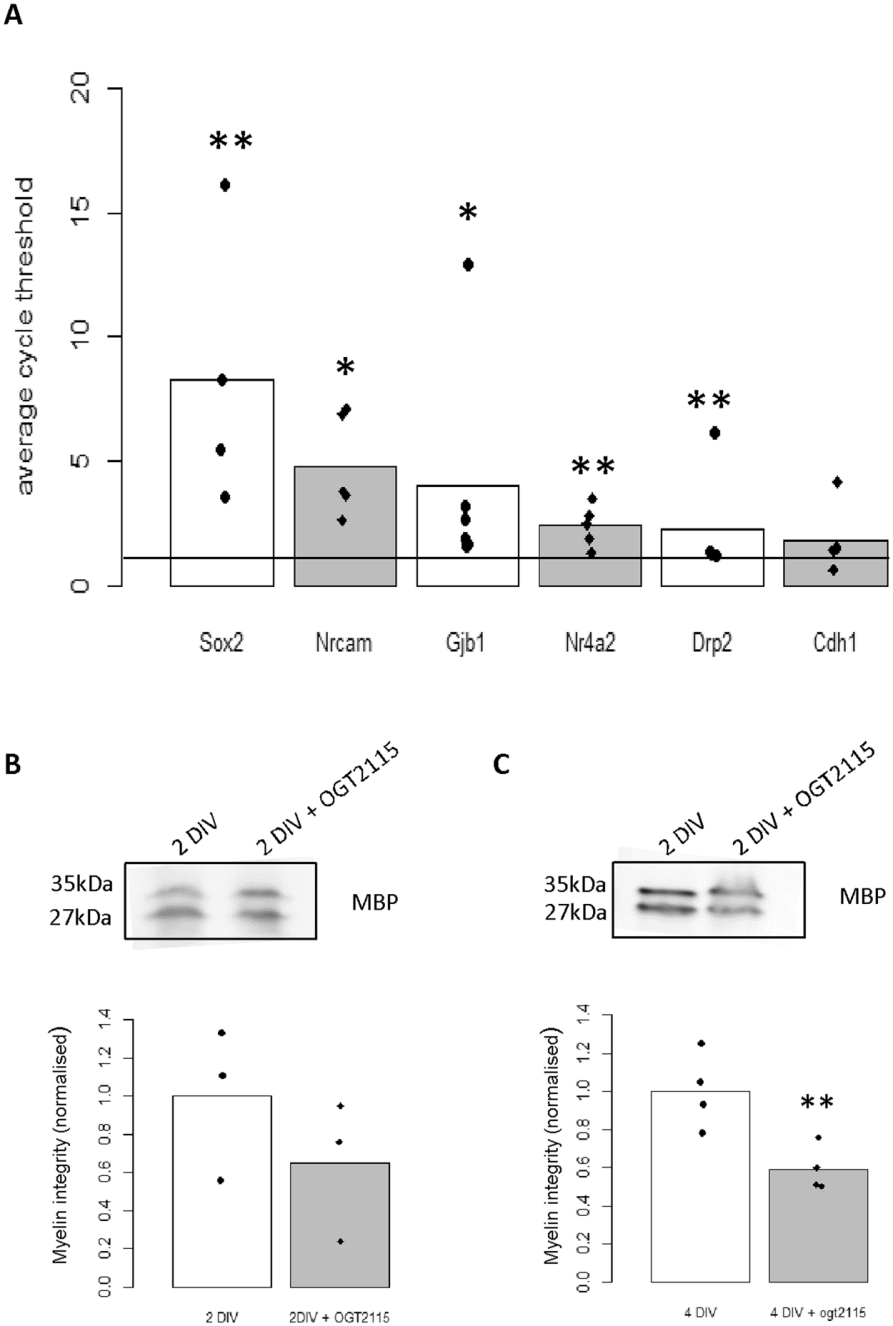
Heparanase regulates Schwann cell de-differentiation markers. (**A**) RT-qPCR for *ex vivo* SN comparing 2 DIV to 2 DIV + OGT2115. Gjb1 (p = 0.02, n = 6), Drp2 (0.003, n = 5), Nrcam (p = 0.016, n = 5), Sox2 (p = 0.011, n = 4), Nr4a2 (p = 0.004, n = 5), Cdh1 (p = 0.6, n = 7). Line refers to 1 i.e. no difference between control and treatment. (**B**) Representative Western blot for MBP in the *ex vivo* SN after 2 DIV compared to 2 DIV + OGT2115. Quantification shows no significant difference to control (p = 0.33, n = 3). (**C**) Representative Western blot for MBP in the *ex vivo* SN after 4 DIV compared to 4 DIV + OGT2115. Quantification shows a significant decrease in MBP after OGT2115 treatment (p = 0.003, n = 3). For all Western blots shown in (**B**–**C**), the full length blots are available in supplementary section Fig. S4. In all cases amido black whole protein stain was used as a loading control, representative blots are available in supplementary section Fig. S4. Students T test or one sample T test was used for all statistical analysis.

To validate the DE genes of these 21 genes we carried out RT-qPCR of the *ex vivo* SN for 6 selected genes. Nrcam, Gjb1 and Sox2 were significantly DE after injury while Cdh1, Drp2 and Nr4a2 were not statistically DE (Supplementary Fig. 3). These genes were then investigated after Hpse inhibition following which Gjb1 (p = 0.02), Drp2 (p = 0.003), Nrcam (p = 00016), Sox2 (p = 0.011), Nr4a2 (p = 0.004) but not Cdh1 (p = 0.06) were significantly DE (Fig. 6A). Sox2 showed the largest differential expression after OGT2115 treatment. As Sox2 has been previously shown to promote Schwann cell de-differentiation^35,36^ we hypothesised that the increase in Sox2 mRNA after Hpse inhibition is acting to promote Schwann cell de-differentiation. In support of this dedifferentiation hypothesis we observed a gradual decline in MBP expression on Western blot with Hpse inhibition (Fig. 6B,C, 2 DIV p = 0.3, 4 DIV p = 0.003).

## Discussion

Whilst extensive research into the mechanisms of axonal degeneration has been conducted, the molecular pathways involved remain unclarified with no specific therapeutics identified. Here we highlight a role for HS in modulating peripheral axon degeneration following injury and identify the regulation of HS signalling as an important pathway for protecting peripheral nerve axon degeneration. HS have the ability to regulate multiple signalling pathways^16^ and whilst they have been identified as effective therapeutic targets in several diseases, including cancer^37^, Alzheimer’s disease^38^ and diabetes^39^, their role in peripheral nerve injury has not been explored. We have identified Hpse, the enzyme that modulates HS signalling, as a protector of axon degeneration. Our data indicates that Hpse is protective, and Hpse inhibition accentuates axon degeneration as a consequence of β-catenin signalling and Schwann cell de-differentiation.

**Figure 7:**
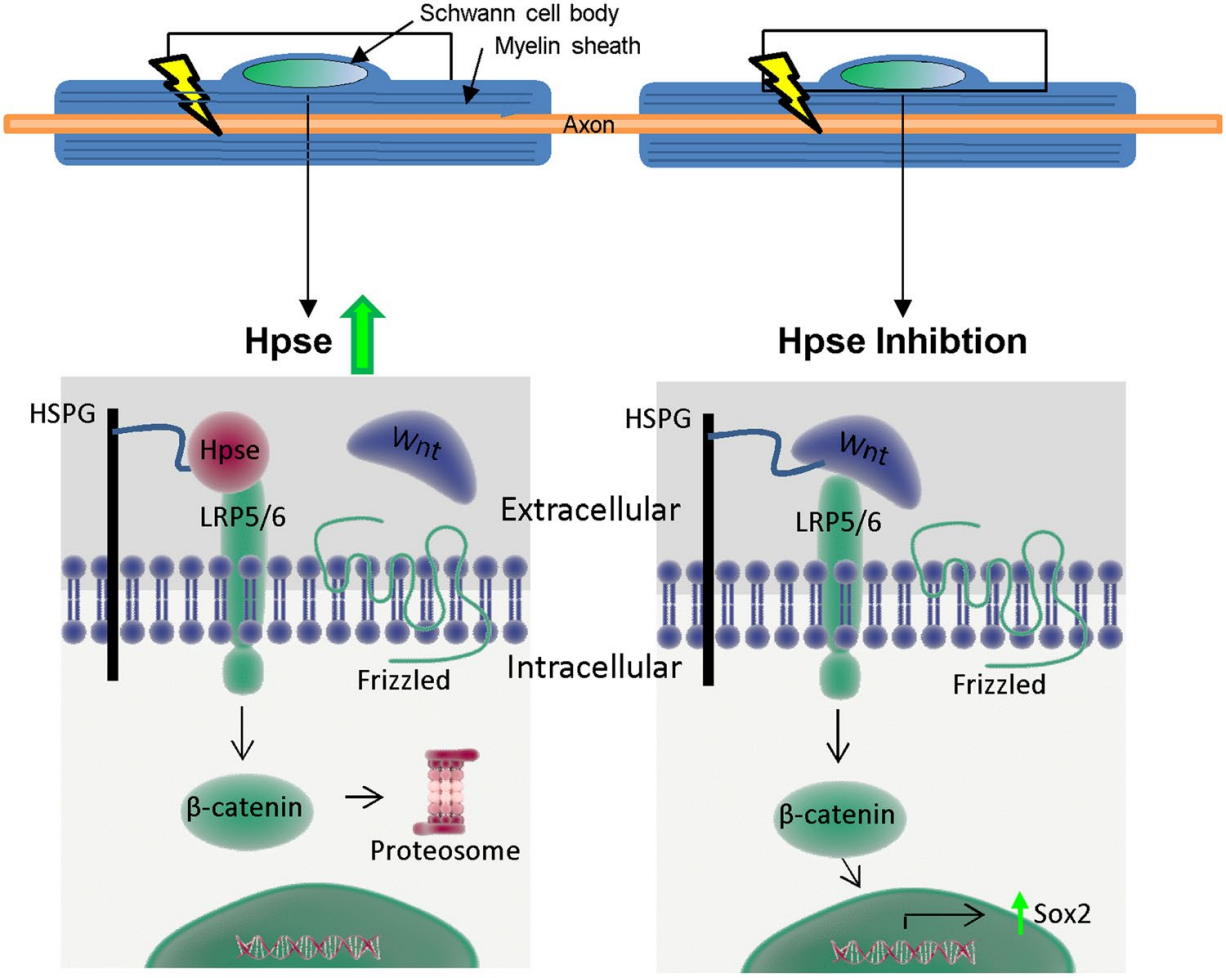
Hypothesis for the regulation of β-catenin by Hpse, in Schwann cells. The square indicates the region of the Schwann cell body and the abaxonal myelin sheath layers magnified in the lower schematics. When Hpse is up-regulated (after injury) the enzyme cleaves HS which decreases the likelihood of Wnt ligands binding to their receptor. This absence in signal results in the proteosomal degradation of β-catenin. When Hpse is inhibited by OGT2115 (during injury) the likelihood of the Wnt ligands binding to their receptor increases which stabilises β-catenin. β-catenin is then able to enter the nucleus and transcribe genes such as Sox2 which will promote SC de-differentiation and, we hypothesise, promote axon degeneration.

Our findings demonstrating that Hpse attenuates axon degeneration in the *ex vivo* SN explant model is the first functionally relevant report of Hpse activity in the PNS. Whilst glia in the CNS have been shown to express Hpse^26,40^, this is the first study to identify its expression in Schwann cells. Hpse has previously been shown to protect against axon degeneration in Alzheimer’s disease models, where it inhibits HS mediated amyloid-β deposition^41^. The only known role for Hpse2 (an in-active splice variant of Hpse) specifically in the PNS is in the development of urofacial syndrome, where Hpse2 knock-out perturbs the correct development of motor neurones via fibroblast growth factor (FGF) mediated gene transcription^42^. Hpse has also been shown to promote neurite out growth via regulation of nerve growth factor (NGF) signalling^25^ and HS plays an important role in axon guidance^43^. Hpse activity also modulates neuroinflammatory effects on macrophage activation (via c-fos induced cytokine expression)^44^, the recruitment of immune cells to the site of injury (via remodelling of the extracellular matrix)^45^, and autophagy^46^. Whether Hpse affects the immune response after peripheral nerve injury, as well as its role during regeneration of the proximal stump, has not been investigated herein. In the SN, HS are expressed on the abaxonal myelin sheath and we show that their remodelling via the up-regulation of Hpse after injury inhibits β-catenin signalling (Fig. 7). As such inhibiting Hpse, which accelerates axon degeneration, also promotes β-catenin signalling.

The link between β-catenin and axonal degeneration in the *ex vivo* SN explant model is previously unidentified. β-catenin has also been shown to promote axon loss in a PD model and conversely to protect against axon degeneration in AD models^46,47^. Whilst the mechanisms behind its negative role in PD are unclear, in AD it regulates mitochondrial damage by closing the mitochondrial permeability transition pore (mPTP)^47^, which has also been shown to play a fundamental role in SN injury^48^. Canonical Wnt signalling is a ubiquitous and important pathway in many systems. In Schwann cells, β-catenin is a fundamental regulator of development and subsequent radial sorting^49^. β-catenin has also been shown to promote Schwann cell myelination by activating the transcription of myelin proteins^50^. Microarray analysis has implicated Wnt signalling in SN injury previously^51^, but was not subsequently explored. From the current data we hypothesise a role for β-catenin in injury induced de-differentiation of Schwann cells.

We also hypothesise that Hpse inhibition promotes Schwann cell de-differentiation mediated by a β-catenin/Sox2 axis. β-catenin has previously been shown to up-regulate the transcription of Sox2 in other contexts^52^. It is known that Sox2 plays an important role in inhibiting Schwann cell myelination and promoting de-differentiation and proliferation^35,36^. Moreover, Schwann cells de-differentiate after injury into a more immature progenitor like state^9^. Here we show that Hpse inhibition increases Sox2 transcription, which in turn promotes Schwann cell de-differentiation. Myelinating Schwann cells are tightly coupled to axons and provide metabolites, the disruption of which, specifically in Schwann cells, can cause age dependent axon degeneratio^10,12^. We therefore hypothesise that Schwann cell de-differentiation disrupts this axo-glia symbiosis, which in turn accelerates axon degeneration; as has been hypothesised recently^53^. However it is also plausible that heparanase acts by releasing growth factors from the extracellular matrix (ECM)^37^, several of which have been implicated as therapeutics in peripheral neuropathies^54^; including nerve growth factor (NGF), which protected small fibre degeneration in a diabetic mouse model^55^. Thus, Hpse mode of action may have several mechanisms of action including via β-catenin signalling, and potentially also via the release of growth factors. Hpse is already a therapeutic target in other diseases, and we highlight a new application following peripheral nerve injury mediated via inhibiting a β-catenin/Sox2 signalling axis, that in turn attenuates Schwann cell de-differentiation and axon degeneration.

## Methods

### Bioinformatics

Previously published microarrays were accessed from NCBI’s gene expression omnibus (GEO) microarray depository: GSE22291, GSE38693, and GSE58982^28,56,57^.

Microarrays were analysed using GEO2R, the affiliated tool for the GEO depository that uses the Bioconductor packages in R^58^. Heat maps were generated using the heatmaps2 function in ggplot for R. Volcano plots were generated using the standard plot function in R. Annokey online was run using python version 2.7.12 via the macOS terminal^33^. Enriched transcription factor prediction was performed using the molecular signatures database^59^. The dplyr package in R was used to filter and sort microarray results. R version 3.3.2 using the integrated development environment (IDE) RStudio. DE genes are those with a log2fc <1.5 or log2fc <−1.5 and an adjusted p value < 0.05. For the unbiased identification of genes potentially important in sciatic nerve injury they were selected if DE in WT injury and equally and oppositely expressed in Wld^s^ injury compared to WT. This strict cut off was used instead of simply selecting genes DE in WT and not DE expressed in Wld^s^ in an attempt to reduce the number of potential candidates.

### Animals and ethical considerations

Adult Sprague Dawley (SD) rats from Harlan laboratories were used for *ex vivo* sciatic nerve explants. For triangularis sterni (TS) neuromuscular preparations, homozygous and heterozygous (F1) double-fluorescent adult B6.Cg-Tg(Thy1-CFP/S100B-GFP) mice, which express intracytosolic CFP in their peripheral motor and sensory axons and intracytosolic GFP in their Schwann cells were used as previously described^60^. All experiments were performed in accordance with a licence approved and granted by the United Kingdom Home Office and conformed to University of Glasgow institutional guidelines. Procedures complied with relevant guidelines outlined in the revised Animals (Scientific Procedures) Act 1986.

### *Ex vivo* explant cultures of sciatic nerve degeneration and triangularis sterni preparations

The *ex vivo* sciatic nerve degeneration assay was conducted as previously described^48^. Briefly both sciatic nerves were dissected from adult SD rats and cut into four equal segments. The segments were cultured in 24 well plates with 500 µl of Neurobasal media (ThermoFisher Scientific) supplemented with B27 (ThermoFisher Scientific), for the indicated number of days. Triangularis sterni (TS) muscles were dissected from the rib cage of B6.Cg-Tg mice pinned out flat as previously described^61^ and maintained in Ringers solution at 4 °C for 19 hrs. The Hpse inhibitor OGT2115 (Tocris Bioscience) was used at 8 μM^62^. There is believed to be only one enzymatically active Hpse in mammals, and OGT2115 is the only commercially available inhibitor, its precise mechanism of action is not known. XAV939 the β-catenin inhibitor was used (Tocris Biosciences) at 40 μM^63^. Both inhibitors were dissolved in DMSO and all controls were treated with DMSO only. Inhibitors were immediately added to the excised sciatic nerves or TS muscle.

### Purified Schwann cells

Schwann cells were harvested and purified according to O’Neill *et al*.^13^, modified from Raff *et al*.^64^. Briefly Schwann cells were purified from P7 sciatic nerves of F344‐Tg(UBC‐EGFP)F455Rrrc (RRRC, Rat Resource and Research Centre) and cultured in DMEM with 5% FBS and supplemented with 0.5 μM forskolin (Sigma-Aldrich) and 50 nl/ml heregulin β1 (R&D Systems). Schwann cells were purified using an immunomagnetic positive selection kit (EasySep, Stem Cell Technologies), with the antibody p75^NTR^ (Abcam).

### Immunofluorescence and histology

*Ex vivo* SN tissue was fixed in 4% paraformaldehyde (1xPBS, pH 7.4) for 1 hr, washed in PBS and placed in 30% sucrose solution for 1hr. The nerve segment was then cut in half with a razor blade and frozen in OCT (Tissue-TEK) and transverse sections cut with a cryostat. For each experiment one nerve was used as control and the other for treatment. Two contralateral segments from each sciatic nerve were used per condition. Each nerve segment was cut into 9 sections with a 100 μM gap between each three. Tissue was permeabilised for internal antigens using ethanol at −20 °C for 20 min, prior to primary antibody(s) incubation o/n at 4 °C. After washing the secondary antibody was incubated for 1 hr at room temp (RT) washed again and mounted in Vectashield (Vector Labaratories). The number of axons per region of interest was manually counted in Fiji using the cell counter plugin with SMI31 staining, for a set area of interest (40.4mm^2^). All exposure settings on a Leica DFC350 FX microscope were kept constant between images.

Immunocytochemistry for the NMJ *ex vivo* preparation was performed similarly to the *ex vivo* sciatic carried out by counting whether 100 NMJ (identified by post-synaptic α-bungarotoxin that binds nAChR positivity) were innervated by SMI3I positive axons or not (which we define as end plate occupancy). Histology of sciatic nerve explants was performed by first perfusing the tissue with 5% glutaldehyde/4% PFA before resin embedding according to^65^. Semi-thin sections were stained with methylene blue/azurII and degenerating axons were counted as previously.

### RT-qPCR

TRIzol (Invitrogen) was used to extract RNA with a purelink micro RNA extraction kit (Abcam). Quantitech reverse transcriptase kit (Qiagen) was then used to make cDNA from the RNA. cDNA was amplified using Quantifast Sybr green (Qiagen) and quantified using the 7500 fast real-time PCR machine. Fold changes were calculated using the ΔΔct method. Primers were designed using the pick primers function for NCBI and made by integrated DNA technologies (IDT). Housekeeping primers were used and made according to^66^. Table 1 illustrates primers used for RT-PCR.

**Table 1:** Primers for RT-PCR.

**Gene**	**Forward primer**	**Reverse primer**
Hpse	CAATGATAT TTGCGGGTCTG	TGCGTT TTGGAA AGCTGACT
CtsL	AATGGGGAGTACAGCAACGG	TCCTCATTGGTCATGTCACCG
Galns	GCTCATGGACGATATGGGGT	TGTGTAGAAGCCATTGCGGA
Gpc1	GTATCGATGACCACTTCCAGC	AGAGACGCAGCTCAGCATAC
Gpc6	ATGCAGCACAGTGTCACAGA	TGTGTCCATTCCAGCACTCC
Wif1	AAAGCAAGTCTGTCTGCGAG	CGACACTGGCATTTGTTGGG
Rspo1	GCAGTCCTGCACAATGTGAAAT	ATCCCTTCCGGAAACCACAC
Cdh1	TTCAACCCAAGCACGTACCA	TGTACACAGCATTCCACGCT
Drp2	CGACTGCAGTGAGACACCT	TGCTCAATTCGAGAGTGTGTG
Gjb1	CTTGCTCAGTGGCGTGAATC	ATGACGGACAGCCATACTCG
Nr4a2	GGTTTCTTTAAGCGCACGGT	TAAACTGTCCGTGCGAACCA
Nrcam	CGCTCTGGATGTTCCTCTTGA	GTCCAGGAAAAGCTTGGAGGA
Sox2	CTCTGTGGTCAAGTCCGAGG	ATGCTGATCATGTCCCGGAG
Ankrd27	TGCAGAGCAGGACTCAAAGAT	GCAGGTTGTACATTTTGGGGTT

### Western blot

Sciatic nerve segments were lysed and homogenised in Cell Lytic MT lysis buffer with protease inhibitor cocktail (Sigma-Aldrich). Protein samples (20 μg) were treated with reducing agent and sample buffer (Invitrogen) before 10 min incubation at 90 °C. Once cooled samples were run on SDS-PAGE gels (Invitrogen, NuPAGE 4–15% Bis-Tris gel) and transferred to nitrocellulose using an iblot (Invitrogen). The Western blot was performed using standard techniques, visualised and captured using the Azure C500 (Azure Biosystems) and densitometry analysis was performed using Fiji. Whole protein was used as a loading control with the amido black stain (Sigma) where densitometry analysis for each entire lane was used to normalise in Fiji, after being captured using the AzureC500 (Azure Biosystems)^67^.

### Primary/Secondary antibodies

The following antibodies were used for immunofluorescence (IF) and/or Western blot analysis: SMI31 – 1:1000 for WB and IF (Millipore, NE1022). MBP – 1:500 for WB and IF (Millipore, MAB382). β-catenin WB 1:500 (BD Biosciences, 610153). Active β-catenin (clone 8E7) (Millipore). α-bungarotoxin (Btx: to visualise NMJ, Invitrogen). F69-3G10 – 1:500 for WB (referred to as 3G10 in results) (Amsbio). F58-10E4 – 1:100 for IF (referred to as 10EA in results)(Amsbio). Sox2 – 1:1000 for Western blot (Abcam). WB secondary: Mouse IgG HRP-linked (GE Healthcare, NXA931). IF secondary: Alexa Fluor mouse IgG1, IgG2a (ThermoFischer Scientific, A-21121).

### Statistics

An unpaired Student’s T test was performed for statistical analysis when comparing two conditions and a one-sample T test was used for RT-qPCR analysis. For multiple comparisons a one-way ANNOVA with Tukey honestly significant difference (HSD) post hoc test was performed. p < 0.05 and a confidence interval that did not cross 0 was considered statistically significant. For immunohistochemical analysis and Western blots of tissue n = 3–4 were performed. For RT-qPCR n = 3–6 were performed. These n numbers were based on previous experience with these methods in the laboratory.

## Electronic supplementary material

supplementary information

## References

[1] Perry VH, Lunn ER, Brown MC, Cahusac S, Gordon S (1990). Evidence that the Rate of Wallerian Degeneration is Controlled by a Single Autosomal Dominant Gene. Eur. J. Neurosci..

[2] Wang MS, Davis AA, Culver DG, Glass JD (2002). Wlds mice are resistant to paclitaxel (taxol) neuropathy. Ann. Neurol..

[3] Watanabe M, Tsukiyama T, Hatakeyama S (2007). Protection of vincristine-induced neuropathy by WldS expression and the independence of the activity of Nmnat1. Neurosci. Lett..

[4] Zhu SS (2011). WldS protects against peripheral neuropathy and retinopathy in an experimental model of diabetes in mice. Diabetologia.

[5] Court FA, Coleman MP (2012). Mitochondria as a central sensor for axonal degenerative stimuli. Trends Neurosci..

[6] George EB, Glass JD, Griffin JW (1995). Axotomy-induced axonal degeneration is mediated by calcium influx through ion-specific channels. J. Neurosci. Off. J. Soc. Neurosci..

[7] Ma M (2013). Calpains mediate axonal cytoskeleton disintegration during Wallerian degeneration. Neurobiol. Dis..

[8] Shen H, Hyrc KL, Goldberg MP (2013). Maintaining energy homeostasis is an essential component of WldS-mediated axon protection. Neurobiol. Dis..

[9] Jessen KR, Mirsky R (2016). The repair Schwann cell and its function in regenerating nerves. J. Physiol..

[10] Beirowski B (2014). Metabolic regulator LKB1 is crucial for Schwann cell-mediated axon maintenance. Nat. Neurosci..

[11] Bunge RP (1994). The role of the Schwann cell in trophic support and regeneration. J. Neurol..

[12] Viader A (2011). Schwann cell mitochondrial metabolism supports long-term axonal survival and peripheral nerve function. J. Neurosci. Off. J. Soc. Neurosci..

[13] O’Neill P (2017). Sulfatase-mediated manipulation of the astrocyte-Schwann cell interface. Glia.

[14] Higginson JR (2012). Differential sulfation remodelling of heparan sulfate by extracellular 6-O-sulfatases regulates fibroblast growth factor-induced boundary formation by glial cells: implications for glial cell transplantation. J. Neurosci. Off. J. Soc. Neurosci..

[15] Eshed-Eisenbach Y, Gordon A, Sukhanov N, Peles E (2016). Specific inhibition of secreted NRG1 types I-II by heparin enhances Schwann Cell myelination. Glia.

[16] Sarrazin, S., Lamanna, W. C. & Esko, J. D. Heparan Sulfate Proteoglycans. *Cold Spring Harb. Perspect. Biol*. **3** (2011). 10.1101/cshperspect.a004952 PMC3119907 21690215

[17] Yan, D. & Lin, X. Shaping Morphogen Gradients by Proteoglycans. *Cold Spring Harb. Perspect. Biol*. **1** (2009). 10.1101/cshperspect.a002493 PMC2773635 20066107

[18] Binari RC (1997). Genetic evidence that heparin-like glycosaminoglycans are involved in wingless signaling. Dev. Camb. Engl..

[19] Fuerer C, Habib SJ, Nusse R (2010). A study on the interactions between heparan sulfate proteoglycans and Wnt proteins. Dev. Dyn. Off. Publ. Am. Assoc. Anat..

[20] Rapraeger AC, Krufka A, Olwin BB (1991). Requirement of heparan sulfate for bFGF-mediated fibroblast growth and myoblast differentiation. Science.

[21] Schlessinger J, Lax I, Lemmon M (1995). Regulation of growth factor activation by proteoglycans: what is the role of the low affinity receptors?. Cell.

[22] Vlodavsky I, Ilan N, Naggi A, Casu B (2007). Heparanase: structure, biological functions, and inhibition by heparin-derived mimetics of heparan sulfate. Curr. Pharm. Des..

[23] McKenzie E (2003). Biochemical characterization of the active heterodimer form of human heparanase (Hpa1) protein expressed in insect cells. Biochem. J..

[24] Changyaleket B, Deliu Z, Chignalia AZ, Feinstein DL (2017). Heparanase: Potential roles in multiple sclerosis. J. Neuroimmunol..

[25] Cui H (2011). Heparanase enhances nerve-growth-factor-induced PC12 cell neuritogenesis via the p38 MAPK pathway. Biochem. J..

[26] Marchetti D, Li J, Shen R (2000). Astrocytes contribute to the brain-metastatic specificity of melanoma cells by producing heparanase. Cancer Res..

[27] Navarro FP (2008). Brain heparanase expression is up-regulated during postnatal development and hypoxia-induced neovascularization in adult rats. J. Neurochem..

[28] Arthur-Farraj PJ (2012). c-Jun reprograms Schwann cells of injured nerves to generate a repair cell essential for regeneration. Neuron.

[29] Abboud-Jarrous G (2008). Cathepsin L Is Responsible for Processing and Activation of Proheparanase through Multiple Cleavages of a Linker Segment. J. Biol. Chem..

[30] Rivera-Colón Y, Schutsky EK, Kita AZ, Garman SC (2012). The Structure of Human GALNS Reveals the Molecular Basis for Mucopolysaccharidosis IV A. J. Mol. Biol..

[31] Tsao JW, George EB, Griffin JW (1999). Temperature Modulation Reveals Three Distinct Stages of Wallerian Degeneration. J. Neurosci..

[32] Han J, Mandal AK, Hiebert LM (2005). Endothelial cell injury by high glucose and heparanase is prevented by insulin, heparin and basic fibroblast growth factor. Cardiovasc. Diabetol..

[33] Park DJ, Nguyen-Dumont T, Kang S, Verspoor K, Pope BJ (2014). Annokey: an annotation tool based on key term search of the NCBI Entrez Gene database. Source Code Biol. Med..

[34] MacDonald BT, Tamai K, He X (2009). Wnt/β-catenin signaling: components, mechanisms, and diseases. Dev. Cell.

[35] Le N (2005). Analysis of congenital hypomyelinating Egr2Lo/Lo nerves identifies Sox2 as an inhibitor of Schwann cell differentiation and myelination. Proc. Natl. Acad. Sci. USA.

[36] Roberts, S. L. *et al*. Sox2 expression in Schwann cells inhibits myelination *in vivo* and induces influx of macrophages to the nerve. *Dev. Camb. Engl*. 10.1242/dev.150656 (2017). 10.1242/dev.150656 PMC5611958 28743796

[37] Ilan N, Elkin M, Vlodavsky I (2006). Regulation, function and clinical significance of heparanase in cancer metastasis and angiogenesis. Int. J. Biochem. Cell Biol..

[38] Lorente-Gea L, García B, Martín C, Quirós LM, Fernández-Vega I (2017). Heparan sulfate proteoglycans and heparanases in Alzheimer’s disease: current outlook and potential therapeutic targets. Neural Regen. Res..

[39] Gil N (2012). Heparanase is essential for the development of diabetic nephropathy in mice. Diabetes.

[40] Zhang Y (2006). Mapping heparanase expression in the spinal cord of adult rats. J. Comp. Neurol..

[41] Roberts NA (2014). Heparanase 2, mutated in urofacial syndrome, mediates peripheral neural development in Xenopus. Hum. Mol. Genet..

[42] Lee J-S, Chien C-B (2004). When sugars guide axons: insights from heparan sulphate proteoglycan mutants. Nat. Rev. Genet..

[43] Gutter-Kapon L (2016). Heparanase is required for activation and function of macrophages. Proc. Natl. Acad. Sci..

[44] Parish CR (2006). The role of heparan sulphate in inflammation. Nat. Rev. Immunol..

[45] Ilan N, Shteingauz A, Vlodavsky I (2015). Function from within: Autophagy induction by HPSE/heparanase—new possibilities for intervention. Autophagy.

[46] DAI T-L (2014). Depletion of canonical Wnt signaling components has a neuroprotective effect on midbrain dopaminergic neurons in an MPTP-induced mouse model of Parkinson’s disease. Exp. Ther. Med..

[47] Arrázola, M. S., Silva-Alvarez, C. & Inestrosa, N. C. How the Wnt signaling pathway protects from neurodegeneration: the mitochondrial scenario. *Front. Cell. Neurosci*. **9** (2015). 10.3389/fncel.2015.00166 PMC4419851 25999816

[48] Barrientos SA (2011). Axonal Degeneration Is Mediated by the Mitochondrial Permeability Transition Pore. J. Neurosci. Off. J. Soc. Neurosci..

[49] Grigoryan T (2013). Wnt/Rspondin/β-catenin signals control axonal sorting and lineage progression in Schwann cell development. Proc. Natl. Acad. Sci. USA.

[50] Tawk M (2011). Wnt/beta-catenin signaling is an essential and direct driver of myelin gene expression and myelinogenesis. J. Neurosci. Off. J. Soc. Neurosci..

[51] Yao D (2012). Gene expression profiling of the rat sciatic nerve in early Wallerian degeneration after injury. Neural Regen. Res..

[52] Van Raay TJ (2005). Frizzled 5 signaling governs the neural potential of progenitors in the developing Xenopus retina. Neuron.

[53] Wong KM, Babetto E, Beirowski B (2017). Axon degeneration: make the Schwann cell great again. Neural Regen. Res..

[54] Apfel SC (1999). Neurotrophic factors in peripheral neuropathies: therapeutic implications. Brain Pathol. Zurich Switz..

[55] Apfel SC, Arezzo JC, Brownlee M, Federoff H, Kessler JA (1994). Nerve growth factor administration protects against experimental diabetic sensory neuropathy. Brain Res..

[56] Barrette B, Calvo E, Vallières N, Lacroix S (2010). Transcriptional profiling of the injured sciatic nerve of mice carrying the Wld(S) mutant gene: identification of genes involved in neuroprotection, neuroinflammation, and nerve regeneration. Brain. Behav. Immun..

[57] Painter MW (2014). Diminished Schwann cell repair responses underlie age-associated impaired axonal regeneration. Neuron.

[58] Barrett T (2013). NCBI GEO: archive for functional genomics data sets—update. Nucleic Acids Res..

[59] Liberzon A (2015). The Molecular Signatures Database Hallmark Gene Set Collection. Cell Syst..

[60] Cunningham ME (2016). Anti-ganglioside antibodies are removed from circulation in mice by neuronal endocytosis. Brain.

[61] McGonigal R (2010). Anti-GD1a antibodies activate complement and calpain to injure distal motor nodes of Ranvier in mice. Brain J. Neurol..

[62] Courtney SM (2005). Furanyl-1,3-thiazol-2-yl and benzoxazol-5-yl acetic acid derivatives: novel classes of heparanase inhibitor. Bioorg. Med. Chem. Lett..

[63] Huang S-MA (2009). Tankyrase inhibition stabilizes axin and antagonizes Wnt signalling. Nature.

[64] Raff MC, Abney E, Brockes JP, Hornby-Smith A (1978). Schwann cell growth factors. Cell.

[65] Griffiths IR, Duncan ID, McCulloch M (1981). Shaking pups: a disorder of central myelination in the spaniel dog. II. Ultrastructural observations on the white matter of the cervical spinal cord. J. Neurocytol..

[66] Gambarotta, G. *et al*. Identification and Validation of Suitable Housekeeping Genes for Normalizing Quantitative Real-Time PCR Assays in Injured Peripheral Nerves. *Plos One* **9** (2014). 10.1371/journal.pone.0105601 PMC4140797 25144298

[67] Aldridge GM, Podrebarac DM, Greenough WT, Weiler IJ (2008). The use of total protein stains as loading controls: an alternative to high-abundance single protein controls in semi-quantitative immunoblotting. J. Neurosci. Methods.

