# Supplementary material for: Heparanase attenuates axon degeneration following sciatic nerve transection
Source: Sci Rep. 2018 Mar 26;8:5219. doi: 10.1038/s41598-018-23070-6 (PMC5980233; doi:10.1038/s41598-018-23070-6)
Supplement: Supplementary file 1 — supplementary information [file 41598_2018_23070_MOESM1_ESM.pdf]

Supplementary Data

Heparanase attenuates axon degeneration following sciatic nerve transection

Michael J. Whitehead, Rhona McGonigal, Hugh J. Willison, Susan C. Barnett*

Institute of Infection, Immunity and Inflammation, College of Medical, Veterinary and Life Sciences, University of Glasgow, 120 University Place, Glasgow G12 8TA, UK

Running title: Haparanase in sciatic nerve injury

*corresponding author:

Professor Sue Barnett

University of Glasgow, Institute of Infection, Immunity and Inflammation, College of Medical, Veterinary and Life Sciences, GBRC, Room B329,

120 University Place, Glasgow, G12 8TA.

Telephone: 44 (0)141 330 8409 E-mail: Susan.Barnett@Glasgow.ac.uk

WT down

3 days	7 days	14 days
Drp2	Drp2	Rgcc
Cdh1	Cdh1	Mfap5
Fam107a	Fam107a	Plscr2
Plekha4	Plekha7	
Kcnk1	Kcnk1	
Fgf1	Fgf1	
Limch1	Limch1	
Col23a1	Col23a1	
Dusp15	Dusp15	
Prss12	Aif1l	
Secisbp2l	Fxyd3	
Paqr6	Tppp3	
Nek1	Zdhhc2	
Ugt8a	Rgcc	
Cadm2	Mbp	
Rimklb	Prkca	
Deptor	Prx	
Mal	Frmd3	
Plekha7	Tmem40	
Pmp2	Tmigd1	
Cmtm5	Drp2	
Nr4a2	Clstn1	
Sema5a	Rgcc	
Satb1	Chn2	
Ndrg1	Slco3a1	
Lrrn1		
Ogn		
Col4a4		
Ctnnal1		
Mpz		
Hmgcs2		
Lmo7		
Cyp39a1		

Up or down at two time points (3&7 days)

Up or down at two time points (7&14 days)

Up regulated DRG

WT up

3 days	7 days	14 days
Fgf5	Fgf5	Fgf5
Nav2	Nav2	Chil3
Hmga2	Clca2	Olig1
Fam102a	Epha5	Mcoln3
Syt4	Tyrp1	Cd300lb
Syt4	Abca1	Fam19a2
Sema4f	Gria1	Ear12
Nptx1	Flrt3	Cil4
Ucn2	Slc26a7	Rnase2a
Lgr6	Gpnmb	Cd5l
Fam102a	Sh3gl3	Il7r
Foxq1	Lrrtm2	
Btc	Rassf3	
Lbh	Tenm3	
Sox2	Epha5	
Tmc7	Tyrp1	
Angptl2	Trpm3	
Gpc1	Ntm	
Bdh1	Btc	
Wfs1	Tyrp1	
Srxn1	Ednrb	
Nrcam		
Sacs		
Dpy19l1		
Camk2d		
Met		
Aldh1a3		
Cgref1		
Tubb3		
Dpy19l1		
Epha5		
Car2		
Clcf1		
Megf10		
Fosl1		
Cgref1		
Aldh1a3		
Gfra1		

Supplementary figure 1: Unbiased screen of genes potentially involved in distal sciatic nerve injury based on previously published microarrays. Previously published microarrays for distal SN injury in WT or Wld^s mice and the DRG after SN injury (as previously described). Genes were selected if they were significantly DE in WT but not in Wld^s or the DRG (refer to methods for analysis).

Down regulated	TCF3	MYOD1	REPIN1	EGR2 *
DRP2	■	■	■	
CADM2	■	■	■	
CRRN1		■		
LRRN1	■		■	
Deptor		■	■	
NR4A2	■	■		■
COL4A4	■	■		
PLEKHA4		■	■	
FGF1	■		■	■
CDH1	■		■	
PRSS12	■			
KCNK1	■			
NRG1			■	■
OGN				■
MAL				■
KCNK1				■
UTG8				■
MPZ				■
LIMCH1				■

Up regulated	LEF1	NF-1	TCF3	EGR2 *
HMAG2	■	■		■
SOX2	■	■		■
GFRAI	■		■	
MET	■			
SYT4	■		■	
GJB1	■		■	
NRCAM	■			
ANGPTL2	■			
MEGF10	■			
NAV2		■	■	■
LGR6		■		
EPHA5				■
FGF5			■	
NPTX1			■	
TMC7			■	

Predicted to transcriptionally regulate
Not predicted to transcriptionally regulate

Supplementary figure 2: Unbiased screen for genes potentially involved in Wallerian degeneration of the distal sciatic nerve after injury show 21 genes are predicted to be transcriptionally regulated by β-catenin. Transcription factor enrichment analysis was performed on genes selected in supplementary Figure 1. 21 genes were predicted to be regulated by LEF1 and TCF3 which are part of the β-catenin signalling pathway. Blue boxes = predicted to be transcriptionally regulated by, white boxes = not predicted to be transcriptionally regulated by.

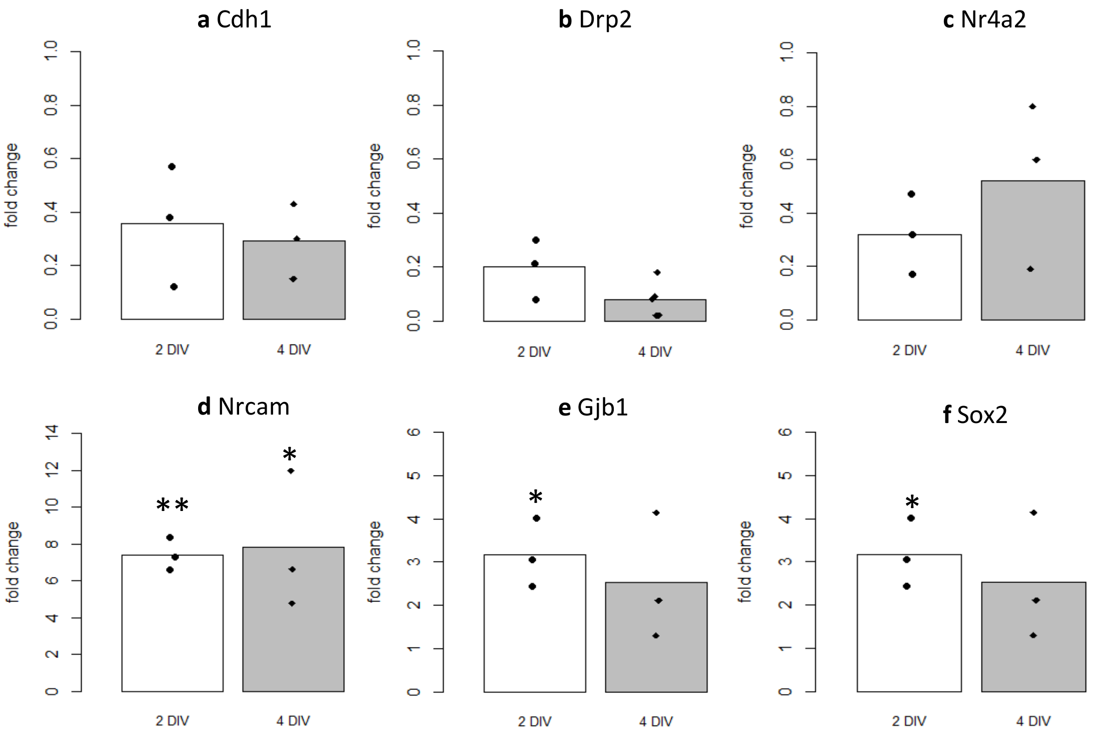

Supplementary figure 3: RT-qPCR validation for selected genes from supplementary figure 1 and 2 using the *ex vivo* sciatic nerve. RT-qPCR was used to validate the observed DE (from the microarrays), in the *ex vivo* SN, for: A) Cdh1 (2 DIV p=0.2 (n=3), 4DIV p=0.09(n=3)), B) Drp2 (2 DIV p=0.2 (n=3), 4DIV p=0.08 (n=5)), C) Nr4a2 (2 DIV p=0.082 (n=3), 4DIV p=0.17 (n=3)), D) Nrcam (2 DIV **p=0.0012 (n=3), 4DIV *p=0.025 (n=3)), E) Gjb1 (2 DIV *p=0.04 (n=3), 4DIV p=0.07 (n=8)), F) Sox2 (2 DIV *p=0.016 (n=3), 4DIV p=0.14 (n=3). A one sample T test was used for statistical analysis.

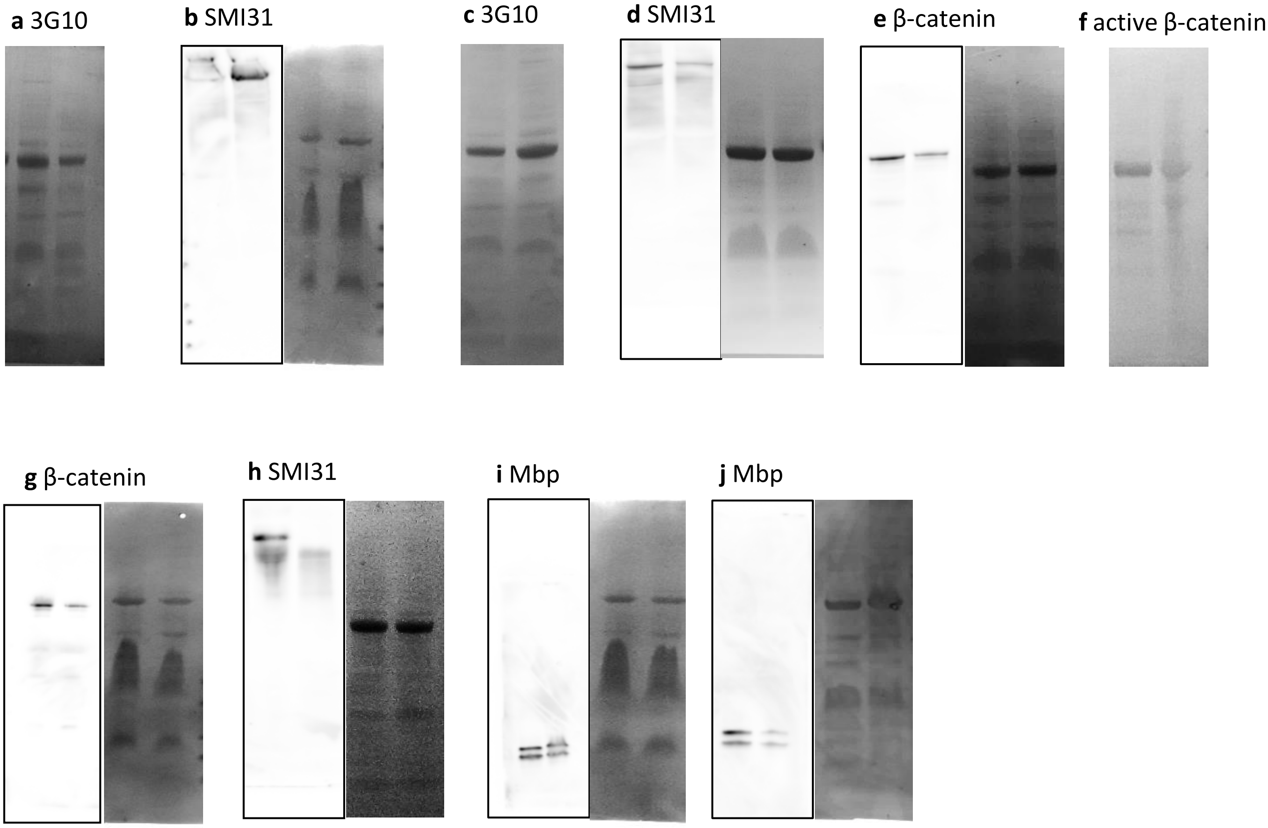

Supplementary figure 4: Full representative Western blots. A) Representative amido black staining Figure 1G. B) Representative full Western blot and amido black staining for Figure 2C. C) Representative amido black staining for figure 2G. D) Representative full Western blot and amido black staining for Figure 1L. E) Representative full Western blot and amido black staining for Figure 5A. F) Representative amido black staining for Figure 5B. G) Representative full Western blot and amido black staining for Figure 5D. H) Representative full Western blot and amido black staining for Figure 5E. I) Representative full Western bot and amido black staining for Figure 6B. J). Representative full Western blot and amido black staining for Figure 6C.
